# Acceleration of Biological Aging and Underestimation of Subjective Age Are Risk Factors for Severe COVID-19

**DOI:** 10.3390/biomedicines9080913

**Published:** 2021-07-29

**Authors:** Tatiana N. Berezina, Stanislav Rybtsov

**Affiliations:** 1 Department of Scientific Basis of Extreme Psychology, Moscow State University of Psychology and Education, Shelepikhinskaya Naberezhnaya, 2A/1, Office 207, 123290 Moscow, Russia; 2 Centre for Regenerative Medicine, Institute for Regeneration and Repair, University of Edinburgh, 5 Little France Drive, Edinburgh EH16 4UU, UK

**Keywords:** disease severity, disease cases, coronavirus, COVID-19, biological aging, subjective aging, psychological aging, pandemic, quarantine, lockdown

## Abstract

In an epidemic, it is important to have methods for reliable and rapid assessment of risk groups for severe forms of the disease for their priority vaccination and for the application of preventive lockdown measures. The aim of this study was to investigate risk factors for severe forms of COVID-19 in adults using indicators of biological and subjective aging. Longitudinal studies evaluated the severity of the disease and the number of cases. Respondents (447) were divided into “working group” and “risk group” (retirees with chronic diseases). During the lockdown period (in mid-2020), accelerated aging was observed in the group of workers (by 3.9–8 years for men and an increase at the tendency level for women). However, the respondents began to feel subjectively younger (by 3.3–7.2 years). In the risk group, there were no deviations from the expected biopsychological aging. The number of cases at the end of 2020 was 31% in workers and 0% in the risk group. Reasonably, the risk group followed the quarantine rules more strictly by 1.5 times. In working men, indicators of relative biological and relative subjective aging (measured in both 2019 and mid-2020) significantly influenced the incidence at the end of 2020. In women, only the indicators obtained in mid-2020 had a significant impact. The relative biological aging of an individual tested in the middle of 2020 had a direct impact on the risk of infection (*p* < 0.05) and on the probability of death (*p* < 0.0001). On the contrary, an increase in the relative subjective (psychological) aging index reduced the risk of infection (at the tendency level, *p* = 0.06) and the risk of death (*p* < 0.0001). Both the risk of infection and the risk of death increased with calendar age at the tendency level. Conclusions: Indicators of individual relative biological and subjective aging affect the probability of getting COVID-19 and its severity. The combination of high indicators of biological aging and underestimated indicators of subjective aging is associated with increased chances of developing severe forms of the disease.

## 1. Introduction

Coronavirus disease (COVID-19) is one of the main threats to public health in different countries worldwide. Therefore, a priority area of medical and psychological research is the development of markers that predict the risk of developing severe forms of COVID-19. Age is considered the main risk factor for coronavirus infection, as well as associated age-related diseases such as diabetes [1], obesity [2], blood coagulation dysfunction [3], and poor health.

This study aimed to identify simple indicators of aging that can be used to predict the probability of severe COVID-19. During a pandemic, it is critical to identify the categories of people most at risk of serious complications or death. Establishing actual risk criteria will facilitate decisions about the need for hospitalisation and about the course of treatment in each specific case and will also provide information for organising temporary shelter and social distancing. Identified indicators can also recommend special attention groups for intensive treatment and priority vaccination during the COVID-19 epidemic.

### 1.1. Approaches to Testing Biological and Psychological Age

There are different approaches to determining an actual person’s age [4,5].

The first approach is calendar age—the actual number of years lived. However, this approach does not always unambiguously reflect the health status of a person and their possible resistance to infectious diseases and other environmental stresses.

**Figure 1:**
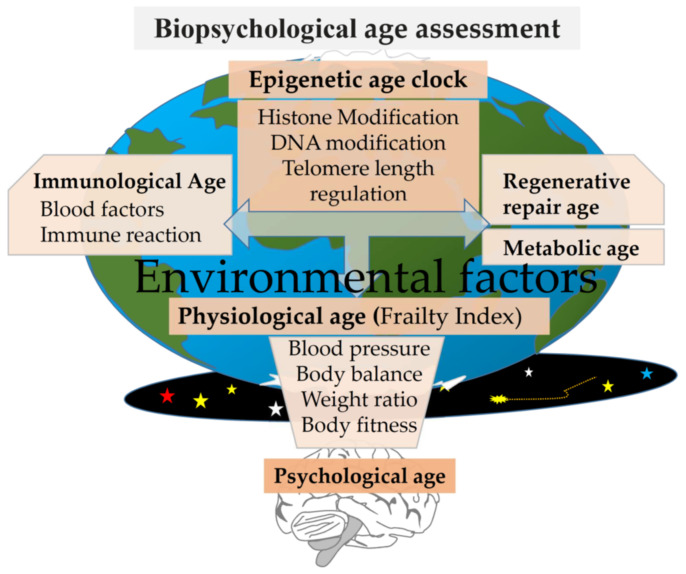
Psychological age and methods for assessing biological age showing the body system’s resistance to various environmental factors (arrows show the hierarchy and interactions of body systems involved/reflecting the aging process).

The second approach is the calculation of the biological age of the body [6,7,8] (Figure 1). Traditionally, biological age is assessed by the morphological, physiological, and functional characteristics of the organism. Biological age can also be compared with the average biological age of a large sample of people of the same calendar age—so called relative biological age [6,9,10,11]. General physiological parameters are often used to estimate biological age, such as vestibular apparatus (body balance), reaction rate, blood sugar level, vascular health, and metabolic measurements [6,7,12,13]. A person’s physical activity, such as the number of steps taken per day, is also considered a valuable indicator of biological age [14]. There are also studies assessing the “age of the brain” [15], the emergence of biomarkers of healthy aging [16], and biomarkers in blood plasma [17,18,19,20,21,22,23].

Biological age can also be calculated using telomerase activity and telomere length [24,25,26] or the degree of DNA or histone modifications [27,28]. 

A significant advance in biological aging analysis was accomplished through the study of DNA modifications that alter gene activity. Methylation of certain DNA sites associated with age-related changes was called the “epigenetic clock” [29]. The predictors were developed based on the analysis of 353 CpG methylation sites in various tissues [29]; later, the number of predictors was reduced to 71 CpG [30]. This method makes it possible to predict life expectancy and other risk factors, including senile diseases [31,32]. During further research, the number of methylation markers predicting all-cause mortality was reduced down to 10 [33]. To improve the prediction accuracy, the expression profiles of genes associated with inflammation, DNA damage, and transcriptional activity were introduced into the model [28].

A recently developed integral model includes, along with the analysis of DNA methylation, the concentrations of several important blood plasma markers of inflammation, cellular stress, and indicators of cell senescence. This approach has increased the accuracy of predicting life expectancy, the probability of senile diseases, and a predisposition to type 2 diabetes. This model shows changes in lifestyle and even the level of education [34]. In recent studies, an index of increasing instability of genetic regulation has also predicted the theoretical maximum life expectancy of humans [35]. 

However, studies of DNA modifications remain rather complex and time-consuming and require a significant amount of donor material for research; therefore, they cannot be massively introduced in clinics.

To assess health status and age-related changes, the Frailty Index (F-Index) is often used. Usually, the F-index combines simple integral indicators of aging biomarkers, physiological state, and physical activity [35,36,37,38]. Five key indicators determine the frailty phenotype: slowness, weakness, physical inactivity, exhaustion, and weight loss, as well as markers of the cardiovascular and respiratory systems [39]. We refer here the integral Frailty Index as “biological age” (see Materials and Methods chapter) [40]. There are also many options for calculating the biological age/Frailty Index, and almost all of them include indicators of the cardiovascular system, respiratory system, body weight, static balancing, and self-assessment of health [13].

The third approach is the assessment of individual subjective (psychological) age (Figure 1) [8,41].

### 1.2. Resistance to COVID-19 Correlates with Biological Age

Many studies point to a correlation between the severity of COVID-19 and calendar age, which, in turn, is associated with a variation in the concentration and diversity of immunity factors in older age groups [22,42]. According to data from China, older people, especially those with dangerous comorbidities, are at a higher risk of serious illness and death associated with COVID-19 than younger people [43]. In the United States, COVID-19 cases from 12 February 12 to 16 March 2020 and disease severity (hospitalisation, admission to intensive care, and death) were analysed by age group. As of 16 March, 4226 cases of COVID-19 have been reported in the US, with multiple cases reported among older adults living in long-term care facilities. Overall, 31% of cases, 53% of hospitalisations, and 80% of deaths associated with COVID-19 were in adults aged 65 and over, with the highest percentage of severe outcomes among people aged 85 and over [44].

However, it has recently been shown that the severity of the disease is more strongly associated with biological rather than calendar age [45,46]. In that study, the epigenetic, glycan clock was used to estimate biological age. Glycans are carbohydrate polymers that regulate immunity and many other processes; the composition of glycans changes with age, especially in the age groups most susceptible to SARS-CoV2 [47]. Thus, the correlation between this measurement of biological age and the severity of COVID-19 is still not entirely clear.

American researchers used a new epigenetic biomarker of aging, “DNAm PhenoAge”, which includes calendar age and nine clinical chemical biomarkers. It has previously been shown to be effective in predicting various outcomes of aging, including all-cause mortality, cancer, health-span, physical functioning, and Alzheimer’s disease [28]. Other authors looked at biological age of 347,751 participants from a large cohort in the United Kingdom (UK Biobank) recruited between 2006 and 2010 and compared disease diagnoses (up to 2017), mortality data (up to 2020), and UK national COVID-19 test results (till 31 May 31 2020). They showed that accelerated aging 10–14 years before the onset of the COVID-19 pandemic was associated with a positive test result (OR = 1.15 for 5-year acceleration, 95% CI: 1.08–1.21, *p* = 3.2 × 10^6^) and all-cause mortality with a confirmed COVID-19 test (OR = 1.25, 5-year acceleration, 95% DI: 1.09–1.44, *p* = 0.002) after adjusting for demographic data, including current calendar age and pre-existing diseases or conditions [37].

However, when assessing the biological age by DNA methylation, the effect is observed less often. For example, using four different age predictors, the authors did not observe accelerated aging in the global DNA methylation profiles of blood samples from nine COVID-19 patients [48]. Predictions correlated well with calendar age, while COVID-19 patients even tended to be predicted as younger than expected. In addition, lymphocytes in nineteen COVID-19 patients did not show significantly accelerated telomere shortening. The authors concluded that DNA methylation biomarkers for biological age are not suitable for predicting a high risk of severe COVID-19 infection in elderly patients.

At the same time, indicators of health and individual biomarkers of aging, including those included in the F-Index (such as indicators of the cardiovascular system) can predict the risk of a severe course of COVID-19. It was also shown that the presence of age-related diseases (hypertension, diabetes, or coronary heart disease) can predict the risk of severe forms of COVID-19 [36]. The opposite trend was also found: COVID-19 can cause the appearance of biomarkers of aging and the deterioration of cardiovascular diseases, including myocardial damage, arrhythmias, acute coronary syndrome, and venous thromboembolism [49].

It is assumed that quarantine measures may affect biological age. For example, in Japan, COVID-19 restriction measures have led to an increase in the senility index of the elderly. To assess biological age, Japanese scientists used the Fragility Screening Index (FSI), a questionnaire for the subjective assessment of age-related changes. The participants were 856 uninfected seniors affected by COVID-19 restrictions. The study showed that the introduction of quarantine resulted in a subjective decrease of daily movements, leg muscle strength, and food intake and in general resulted in an increase of the Fragility Index [50].

To our knowledge, there have been no studies on the correlation between subjective age and the risk of severe COVID-19. However, the impact of the consequences of the COVID-19 pandemic on the subjective perception of time is noted [51]. 

The purpose of this research was to study the effect of biological and subjective age indicators on the risk of severe forms of COVID-19 during the 2020 epidemic in a sample in the Russian Federation. The resulting simple indicators can be immediately used to categorise risk groups by age for priority vaccination and priority hospitalisation in case of infection.

## 2. Materials and Methods

In this research, we used several generally accepted indicators as well as indicators and scales developed in the scope of this study.

Biological age (BA) was determined according to V. P. Voitenko [13] (also detailed in [9]). Calculation is based on the indicators of the cardiovascular system (blood pressure); the state of the respiratory system (breath holding on inhale), the musculoskeletal system, and the equilibrium system (static balancing with closed eyes); metabolism (body weight); and psychological indicators (subjective assessment of health).

Expected biological age (EBA) was calculated for the different age groups. This indicator is considered the statistical norm of biological age within a specific age group. It was used to assess the relative aging index. 

The relative biological aging (RBA) index is the difference between biological age and expected biological age (BA-EBA) and allows the assessment of how much older an individual is than their statistical age norm in terms of their health status. Negative values indicate individual youthfulness, while positive values show individual aging in comparison with statistical norm. This is the main indicator used to assess the dynamics of relative aging. 

Subjective psychological age (PA) was measured according to K.A. Abulkhanova and T.N. Berezina [9]. The test participants were asked to evaluate their age on a 100-point scale (from 0 to 100), where 0 points are the age of a new-born baby, and 100 points represents a person who has already achieved everything and will not be able to achieve more. The method is described in detail in [9].

The relative psychological aging (RPA) index is the psychological age minus calendar age (PA-CA). Negative values indicate the person’s perception to be younger than their calendar age. Positive values indicate that the person considers themselves more mature, wise, and successful than other people at that age.

COVID-19 severity scale was scored as 0 points to indicate that the disease is absent; 1 point to indicate a mild form of the disease or the presence of certain symptoms (positive PCR test); 2 points for the average severity of the disease (hospitalisation); 3 points for a severe degree of the disease (staying in the ICU and/or mechanical ventilation); and 4 points for a fatal outcome (cause of death due to COVID-19). 

Quarantine compliance scale was scored as 0 points to indicate that the participant did not comply at all with restrictive quarantine measures; 1 point for minimum compliance (“just leave me alone”); 2 points for met the basic requirements when leaving home; 3 points for met all the requirements when outside; and 4 points for totally complying by complete self-isolation, not leaving home, and limiting contacts.

### Statistical Analysis

One-way analysis of variance (ANOVA) was used to assess the effect of biopsychological age indicators on the risk of COVID-19. The dependent variable was the severity of COVID-19. The indicators of biopsychological age were used as independent variables. To compare the groups with each other, we used the Fisher LSD test, which is part of the analysis of variance. To assess the link between quarantine compliance and the incidence of disease in groups, we used the φ * criterion—Fisher’s angular transformation.

To evaluate the correlation between indicators of biopsychological age in 2019 and 2020, we calculated the Pearson’s linear correlation coefficient.

We used regression analysis (linear regression) to identify the link between the risk of severe forms of COVID-19 and indicators of biopsychological age.

Sample. A total of 447 people aged 35–70 years (306 women) were included in the database of the longitudinal study of retirement reform in Russia. Among them were: (1) working adults—239 people (155 women, average age = 47.7; and 84 men, average age = 51.9), examined at the place of work or study; and (2) retirees with chronic diseases (risk group)—208 people (151 women, average age = 64.7; and 57 men, average age = 66.8). The survey was carried out in an outpatient hospital. Testing was carried out in mid-2019 (the indicators of biopsychological age were assessed), in mid-2020 (the indicators of biopsychological age, the number of cases, the severity of the disease and its outcome were assessed), and at the end of 2020 (the number of cases, the severity of the disease and its outcome were assessed by certified PCR tests). The study was conducted according to the guidelines of the Declaration of Helsinki, and approved by the Ethics Committee of psychological and Interdisciplinary research of the department of extreme psychology of the Moscow State University of Psychology and Education 123290, Moscow, Russia; Shelepikhinskaya Naberezhnaya, 2A/1, protocol No. 59k-03/19 of 23.04.2019 (study of the dynamics of biopsychological age), protocol No. 59k-03/09 from 02.04.2020 (study of psychological factors COVID-19).

## 3. Results

**Figure 2:**
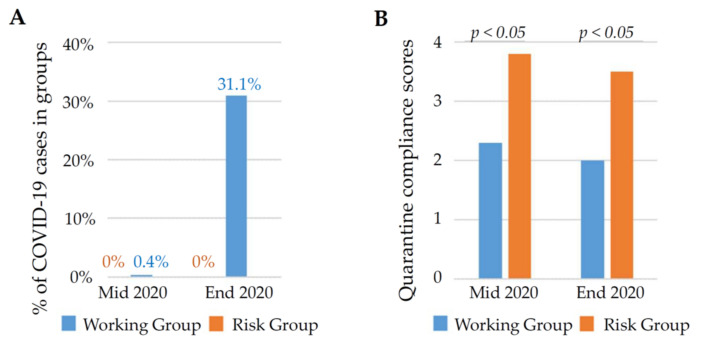
COVID-19 cases (**A**) and compliance with quarantine rules (**B**) in cohort groups during 2020 outbreak. For more information, see Supplementary Table S1.

At the first stage, we studied the disease dynamics in the working group and the risk group. The peak of disease in Russia was in the second half of 2020 (Figure 2). 

In the first half of the year, there were only 0.4% of cases in the working group, reaching 31% by the end of 2020. (Figure 2A, Supplementary Table S1). Interestingly, over the entire observation period, there were no cases in the risk group.

In the first half of the quarantine (until mid-2020), 5% of working adults and 85% people from the risk group totally complied with restrictive quarantine measures (practically without leaving home, 4 points). In the second half, full quarantine was observed (4 points): in the group of working adults—0% of people, in the risk group—52% of people. In general, the risk group complied with restrictions almost 1.7 times better than the working group (Supplementary Table S1). However, there was a slight downward trend in quarantine compliance during the epidemic in both groups, apparently due to general quarantine fatigue (Figure 2B). As a result, in a working group, the incidence of disease was significantly higher than in the risk group that strictly observed quarantine restrictions (φ * Fisher’s test, *p* < 0.001) (Figure 2A).

At the second stage, we studied the dynamics of biopsychological age indicators in 2019 and 2020. We assessed the reliability of changes in biopsychological age indicators during the quarantine using Fisher’s *F* criterion (Table 1). The stability of the indicators of biopsychological age over time was assessed using correlation analysis (Table 2).

As shown in the Table 1, for working women during the quarantine period, the acceleration of biological aging (RBA) occurred at the tendency level, for retirees it did not change. In working men, biological aging accelerated during quarantine (the index of relative biological aging increased and approached the statistical norm), in retirees there were no changes. During the quarantine, the subjective psychological aging (RPA) index increased in both men and women (Table 1).

As shown in the Table 2, indicators of biopsychological age were stable personality features. They were most steady in men (both working adults and retirees), and indicators of psychological age were more stable than biological (Table 2). In the third stage, we examined the effect of biopsychological age indicators on the risk of getting COVID-19. The results of the female sample are presented below in Table 3. The indicators of biopsychological age measured in 2019 did not affect the risk of getting COVID-19 for women. The only trend found was the inverse effect of the relative psychological aging index on disease risk. In 2020, the number of reliable impacts increased. The biological age of a woman, as well as the index of relative biological aging, increased the probability of severe forms of the disease. The risk was also increased by indicators of enhanced pulse pressure and subjective assessment of health, and indicators of body weight and static balancing did not affect the risk of getting the disease. The indicator of relative psychological aging reduced the risk of severe forms of the disease. In other words, women who considered themselves significantly younger than their calendar age had an increased risk of the disease, and women who estimated their age as being close to their calendar age, on the contrary, had a lower risk of developing the disease (Table 3).

The results of the male sample are presented below (Table 4). Some markers of biopsychological age (even measured in 2019) revealed an increased risk of the new infectious disease in men. This was the case for the relative biological aging index (increased risk), while the relative psychological aging index correlated with reduced risk. In 2020, the indicators of subjective health assessment and biological age were added (increased risk), and the relative biological aging index changed to tendency level (Table 4).

Noticeably, the biopsychological age markers in women in 2019 had practically no effect on the risk of disease in 2020, while in men there was such an effect. Most likely, this is because the indicators of biological age in women over the one-year time (including quarantine) turned out to be more variable than in men (see Table 2). Therefore, the indicators of 2019 no longer influenced the risk of the disease, while the indicators of 2020 did since they reflected the current health status. 

**Figure 3:**
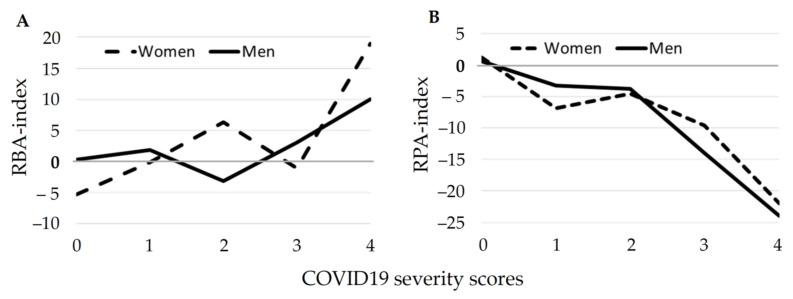
Dynamics of the RBA, relative biological aging index (**A**) and RPA, relative psychological aging index (**B**) in comparison with the severity of the disease in men (solid line) and women (broken line), both from working group. For an explanation of COVID-19 severity scores, see Materials and Methods chapter.

Figure 3 shows the dynamics of the most significant indicators of biopsychological age that reliably affected the onset of severe forms of COVID-19. 

An increase in the relative biological age (RBA) index correlated with the elevated risk of severe forms of COVID-19, both in men (*F* = 1.7104, *p* = 0.17150) and in women (*F* = 1.980, *p* = 0.002) (Figure 3A). An increase of the relative psychological age (RPA) index, on the contrary, reduced the risk of severe forms of COVID-19, both in men (*F* = 2.222, *p* = 0.007) and in women (*F* = 1.837, *p* = 0.004) (Figure 3B).

We also assessed the influence of various indicators of age (1) on the onset of the disease and (2) on the risk of death in patients (in the group of working adults).

As can be seen in Table 3 and Table 4, in the group examined (35–70 years), calendar age affected the onset of the disease and the probability of death only at the tendency level (*p* = 0.1). Biological and psychological age, per se, did not affect the onset of the disease (but biological age increased the probability of death). It was the relative indicators (relative indices, RBA and RPA) that influenced how much a person was older or younger than their calendar age. The older a person was compared with their peers (i.e., the higher their biological age), the higher the probability of the disease onset (*p* = 0.05) and the higher the probability of death of patients (*p* = 0.000). Conversely, the younger a person considered themselves in comparison with their calendar age (an indicator of relative psychological aging), the higher the probability of illness (at the level of tendency, *p* = 0.059) and the probability of death of patients (*p* = 0.00001).

Using regression analysis, we developed an equation for predicting the risk of severe forms of COVID-19 by biological and psychological ages.

COVID-19 Risk Scores = 0.32 + 0.01 × RBA index − 0.003 × RPA index(1)

All addends are significant (*p* < 0.01). The model describes empirical data with validity: *F* (2.236) = 13.137, *p* < 0.0001, R = 0.316. The risk of disease corresponds to our four-point scale, where 1 point is a mild form of the disease, and 4 points is a fatal outcome (see Materials and Methods).

The model can be implemented both for further clinical trials on an expanded sample and for the development of a smartphone application for individual use. 

## 4. Discussion

Biopsychological age includes two components: biological age and subjective psychological age. The biological age of an individual is an integral characteristic of the organism status, health, and its compliance with age norms. Psychological age characterizes a person’s subjective assessment of their life path, achievements, and prospects, as well as their correspondence to calendar age. As our study showed, the risk of severe forms of COVID-19 is influenced not by absolute indicators of biological or psychological age but by relative age. The main risk factor for a person is a significant increase in their biological age with a significant decrease in psychological age relative to calendar age. We believe that such people consider themselves younger than they really are and underestimate the danger of infection.

Severity of COVID19 is age-related; the risk of infection and mortality increases several times with age. For example, according to the National Center for Immunization and Respiratory Diseases (NCIRD) in the United States, the mortality rate is 45 times higher in 30- to 39-year-olds and 8700 times higher in 85+ compared with 5- to 17-year-old children [52]. That is why it is dangerous for a person to consider themselves very young: they begin to believe that they are not endangered by severe forms of COVID-19 and cease to comply with quarantine measures. This is dangerous, especially in a situation where their real health status is even worse than that of their peers (an increase in relative biological aging). 

Our research also showed that strict adherence to quarantine restrictions is a good way to prevent infection. In the Russian risk group (retirees with chronic cardiovascular diseases), no cases of the disease were recorded for six months of observations, in contrast to the younger and healthier group of working adults. This can be explained by the fact that representatives of the risk group very strictly observed quarantine measures, practically never leaving their homes, even contacting the medical workers by phone or being visited at home. Working adults only observed self-isolation measures during the first peak of the disease. However, after the weakening of state requirements for complete self-isolation, they led a more active lifestyle, went to work, and provided livelihoods for the family, including at-risk retired parents. 

We carried out two measurements of the biopsychological age indicators—in 2019 and in 2020. Correlation analyses found that all temporal indicators changed over 1 year to a low degree for the biological age of women, to a medium degree for the biological and psychological age of men, and to a high degree for the psychological age of working women and the relative biological aging of retired men. At the same time, there is also a change in the indicators of the biopsychological age. In women, the indicators of biological age changed the most, while in men, the psychological age indicators changed the most. 

Biopsychological age changes are important as they affect the risk of severe COVID-19. In the group of working women, the indicators of biopsychological age changed more; therefore, their influence on the risk of disease also changed. One of the reasons for the change in indicators may be the quarantine situation. Several studies in Russia have shown that some health indicators improved during the quarantine period (normalisation of blood pressure), others did not change (body weight), and that a significant deterioration occurred only in indicators of physical activity and working capacity (duration of static balancing) [53]. The influence of quarantine (and not disease) on the biological age of the respondents was noted by a number of researchers. In Japan, an increase in biological age, as determined by the Fragility Screening Index (FSI), was revealed in uninfected older people affected by COVID-19 restriction measures [50]. Our study demonstrated that in Russia during the quarantine period, there was a significant growth of biological age only in the group of working men; in the group of working women the increase in biological age was only at the tendency level. The retirees from the risk group did not have an increase in biological age. The indicators of psychological age returned to normal (close to calendar age) during the quarantine. 

Biopsychological age indicators measured in men in 2019 have already had a significant impact on the risk of developing the disease in 2020. Thus, already a year before the pandemic, it was possible to determine the risk group of men by their biopsychological age. These were people whose increased biological age coincided with an underestimated psychological one. For such individuals, the risk of severe forms of the disease, up to and including death, is increased.

In women, only the indicators of biopsychological age, measured in mid-2020, had a significant effect on the infection, and the indicators measured a year before the pandemic had practically no effect on the onset of the disease. In other words, the risk group for women can be determined by their biopsychological age just a few months before the event. This fact has a positive meaning. Biopsychological age is not a calendar age, which over time can only change in one direction, i.e., grow. Both biological and psychological age can change under the influence of external factors or training. For example, a person’s behaviour, lifestyle, and daily routine affect biological age, as measured by metabolic rate [54] and as determined at the chromosomal level [55,56]. Incorporating endurance training into the daily routine of adults affects telomerase activity and telomere length [24] and protects against excessive chromosomal damage [57]. Indeed, many studies have shown a reduction in the number of hospitalisations and the risk of death in COVID-19 patients who had been exercising [58] and were not overweight [59,60]. In addition, aging of the immune system also affects both resistance to infections and the biological age of a person [22,61]. Likewise, the presence of age-related diseases that accelerate biological aging increases the risk of severe consequences of COVID-19 [62].

It can be assumed that activity for normalising biopsychological age indicators, improving health indicators, developing a more adequate attitude towards individual’s age, as well as strict compliance with safety measures, especially for people with complicating diseases, will help reduce the risk of severe forms of COVID-19. In our opinion, this work is not limited to COVID-19 but will have a more general implementation and can apparently be applied to other epidemics of viral infections, as well as for general patient selection for other non-infectious diseases.

### Research Limitations

The “risk group” (retirees with chronic diseases) survey was conducted in an outpatient hospital in Moscow city. All retirees included in research were registered with this clinic for cardiac diseases. Our conclusions about the absence of COVID-19 cases in this group have certain limitations. All participants of this sample lived in Moscow, with families or alone. We did not include any patients from nursing homes. All retirees had an income that provided them with an independent and prosperous lifestyle. For example, many had summer cottages (dachas) where they could self-isolate during the epidemic. 

The “working group” study was also based on the sample of an outpatient hospital in Moscow city. However, information from a number of regions was accumulated here; therefore, data on the risks of illness in adult working people can be interpreted more broadly—for all Russian citizens. Nonetheless, the conclusions on the “working group” refer to working adults (employed) who were directed by their employers to get an annual medical examination. They cannot be attributed to another age group (for example, to young people or to retirees) who are not officially working. In addition, they also can be attributed to the category of people who care about their health.

The study was conducted during the first and second waves of the coronavirus epidemics when people were less aware of the disease and experienced more fear of it. We have not studied the psychological factors affecting the severity of the disease during the third wave of coronavirus.

## 5. Conclusions

Compliance with quarantine measures is an effective way to prevent disease. Representatives of the risk group in Russia (retirees with severe chronic diseases) observed strict self-isolation measures; therefore, not a single case of COVID-19 was found in this group. In the group of working adults for the period of 2020, the disease was registered in 31% of the subjects (2% of deaths).

The probability of contracting a new infectious disease in working adults aged 35–70 years in Russia was most influenced by the relative biological age of the individual; the higher the relative biological aging index was, the higher was the risk of contracting COVID-19 (*p* = 0.05) and the probability of death for the patient (*p* = 0.000). Relative psychological age had an inverse effect on the risk of infection: the higher the index of relative psychological aging, the lower the risk of infection (*p* = 0.06) and the risk of death in those infected (*p* = 0.000). Calendar age increased the risk of infection and the risk of death in our sample but only at the tendency level that did not reach the level of validity.

The most dangerous factor for an individual is a combination of a relative increase in biological age relative to the statistical norm (RBA) and an underestimation of their psychological age relative to calendar age. In this case, the risk of severe forms of an infectious disease increases, up to and including death.

In men, biopsychological age indicators measured in 2019 (the year before the pandemic) had the same effect on the risk of infection as indicators in 2020: relative biological age increased the probability of severe forms of COVID-19, and relative psychological age decreased it. For women, this pattern existed only as a tendency, without reaching the level of validity.

## Figures and Tables

**Table 1:** Dynamics of biopsychological age indicators for men and women in 2019 and 2020.

	Women					Men			
	Working Adults		Retirees from Risk Group			Working Adults		Retirees from Risk Group	
	2019	2020	2019	2020		2019	2020	2019	2020
BA	41.4	43.5	58.6	58.3	BA	47.8	52.4 **	67.7	67.0
RBA	–3.1	–1.5	4.3	4.0	RBA	–2.8	1.1 **	7.7	7.0
PA	50.7	47.7	n/a	n/a	PA	59.9	53.0 *	n/a	n/a
RPA	3.99	0.00*	n/a	n/a	RPA	8.91	1.1 **	n/a	n/a

Comparison with 2019, difference is significant: * *p* < 0.05; ** *p* < 0.01. BA, biological age; RBA (BA-EBA), the relative biological aging index: biological age minus expected biological age; PA, psychological age; RPA (PA-CA), the relative psychological aging index: psychological age minus calendar age; n/a, not available (indicator not measured).

**Table 2:** Coefficients of correlation between indicators of biopsychological age in 2019 and 2020.

	Men		Women	
Correlation between	Working Adults	Retirees from Risk Group	Working Adults	Retirees from Risk Group
BA19 and BA20	0.51 **	0.65 **	0.33 **	0.40 **
RBA19 andRBA20	0.56 **	0.71 **	0.33 **	0.44 **
PA19 andPA20	0.50 **	n/a	0.71 **	n/a
RPA19 and RPA20	0.66 **	n/a	0.63 **	n/a

**- correlation is significant (*p* < 0.01). Year of study indicated (2019–19; 2020–20); BA, biological age; RBA (BA-EBA), the relative biological aging index: biological age minus expected biological age; PA, psychological age; RPA (PA-CA), the relative psychological aging index: psychological age minus calendar age; n/a, not available (indicator not measured).

**Table 3:** Influence of biopsychological age indicators on the occurrence of COVID-19 in women in the working group.

	Data from 2020			Data from 2019		
Indicator	Impact	*F*	*p*	Impact	*F*	*p*
Calendar age	not affected	0.919	0.607	the same as at 2020	-	-
Biological age	increased	1.692	0.014	not affected	0.905	0.643
Relative biological aging index	increased	1.980	0.002	not affected	0.892	0.664
Pulse pressure *	increased	1.776	0.008	not affected	0.955	0.558
Weight	not affected	0.814	0.782	not affected	0.901	0.657
Static balancing	not affected	1.991	0.099	not affected	0.796	0.828
Subjective health assessment	increased	1.682	0.031	not affected	0.836	0.686
Psychological age	not affected	0.840	0.719	not affected	0.776	0.801
Relative psychological aging index	decreased	1.837	0.004	decreasing tendency	1.332	0.105

* Pulse pressure (PP), the difference between systolic blood pressure and diastolic blood pressure (i.e., upper and lower). This indicator is used for women.

**Table 4:** Influence of biopsychological age indicators on the occurrence of COVID-19 in men of the working group.

	Data from 2020			Data from 2019		
Indicator	Impact	*F*	*p*	Impact	*F*	*p*
Calendar age	increased	4.16	0.002	the same as at 2020	-	-
Biological age	increased	2.36	0.004	not affected	0.29	0.828
Relative biological aging index	increasing tendency	1.71	0.171	increased	2.30	0.003
Diastolic blood pressure	not affected	0.73	0.813	not affected	0.74	0.782
Breath holding (BH) *	not affected	1.03	0.462	not affected	0.36	0.777
Static balancing	not affected	0.71	0.837	not affected	0.65	0.911
Subjective health assessment	increased	2.96	0.001	not affected	1.06	0.405
Psychological age	not affected	0.82	0.695	not affected	0.85	0.639
Relative psychological aging index	decreased	2.22	0.007	decreased	2.18	0.007

* BH, duration of Breath Holding after a deep inhale (measured for men).

## Supplementary Materials

The following are available online at https://www.mdpi.com/article/10.3390/biomedicines9080913/s1, Supplementary Table S1: The number of cases, the severity of the disease and compliance with quarantine rules in the middle and end of 2020.
