# Supplementary material for: Acceleration of Biological Aging and Underestimation of Subjective Age Are Risk Factors for Severe COVID-19
Source: Biomedicines. 2021 Jul 29;9(8):913. doi: 10.3390/biomedicines9080913 (PMC8389586; doi:10.3390/biomedicines9080913)
Supplement: Supplementary file 1 [file biomedicines-09-00913-s001.zip › biomedicines-1306584-supplementary.pdf]

Supplementary Table S1. The number of cases, the severity of the disease and compliance with quarantine rules in the middle and end of 2020.

	**Mid-2020**						**The End of 2020**					
	**COVID-19 severity** (% in Group)					Соблюдение карантина	**COVID-19 severity** (% in Group)					Соблюдение карантина
**Severity Groups**	**0**	**1**	**2**	**3**	**4**		**0**	**1**	**2**	**3**	**4**	
Working adults	99,6%	0%	0,4%	0%	0%	**2,3**[$]	69%	12.5%	12,5%	4%	2%	**2,0**[$]
Risk group	100%	0%	0%	0%	0%	**3,8**[$]	100%	0%	0%	0%	0%	**3,5**[$]

[$] quarantine compliance scores
